# Supplementary material for: Newcomer youth’s access to contraception care in Canada: A scoping review of qualitative evidence
Source: PLoS One. 2025 Aug 4;20(8):e0327997. doi: 10.1371/journal.pone.0327997 (PMC12321124; doi:10.1371/journal.pone.0327997)
Supplement: S2 File — (DOCX) [file pone.0327997.s002.docx]

**Medline Search Terms (2010 to 2023)**

1 "emigrants and immigrants"/ or undocumented immigrants/ (15634)

2 Refugees/ (13235)

3 "Transients and Migrants"/ (14261)

4 (immigrant* or immigration or emigrant* or emigration or refugee* or "asylum seeker*" or asylee* or "displaced person*" or "displaced people" or "incomer*" or "in comer*" or "new comer*" or newcomer* or migrant* or resettler*).mp. (99926)

5 1 or 2 or 3 or 4 (99926)

6 contraception/ or contraception, barrier/ or hormonal contraception/ or long-acting reversible contraception/ or natural family planning methods/ (22970)

7 (contracept* or "family planning" or "birth control" or condom or "depot medroxyprogest*" or NET-EN or NET EN or Mesigyna or Cyclofem or "intrauterine system" or "intra-uterine system" or IUS or "intrauterine device" or "intra-uterine device" or IUD or vasectomy or sterilization or "tubal ligation" or "vaginal ring" or cycletel or "cycle-tel" or abstain or abstinen* or "lactational amenorr*").mp. [mp=title, book title, abstract, original title, name of substance word, subject heading word, floating sub-heading word, keyword heading word, organism supplementary concept word, protocol supplementary concept word, rare disease supplementary concept word, unique identifier, synonyms, population supplementary concept word, anatomy supplementary concept word] (208491)

8 sexual health/ or "social determinants of health"/ (8942)

9 health services accessibility/ or access to primary care/ or health equity/ or universal health care/ or "Patient Acceptance of Health Care"/ (138533)

10 sexual health.mp. (14698)

11 family planning.mp. (52289)

12 birth control.mp. (5658)

13 6 or 7 or 8 or 9 or 10 or 11 or 12 (357297)

14 Adolescent/ (2217253)

15 Adolescent Health Services/ (5891)

16 (middle school* or pubescen* or juvenile* or teen* or youth* or high school* or adolesc* or pre-pubesc* or prepubesc* or young adult*).mp. (2937679)

17 14 or 15 or 16 (2937679)

18 5 and 13 and 17 (2431)

19 limit 18 to yr="2010 -Current" (1737)

20 (Canad* or British Columbia or Colombie Britannique or Alberta* or Saskatchewan or Manitoba* or Ontario or Quebec or (New Brunswick not New Jersey) or Nouveau Brunswick or Nova Scotia or Nouvelle Ecosse or Prince Edward Island or Newfoundland or Labrador or Nunavut or NWT or Northwest Territories or Yukon or Nunavik or Inuvialuit).mp,jw,nw. or (Abbotsford or Airdrie or Ajax or Aurora or Barrie or Belleville or Blainville or Brampton or Brantford or Brossard or Burlington or Burnaby or Caledon or Calgary or Cape Breton or Chatham Kent or Chilliwack or Clarington or Coquitlam or Drummondville or Edmonton or Fredericton or Fort McMurray or Gatineau or Granby or Grande Prairie or Sudbury or Guelph or Halton Hills or Iqaluit or Inuvik or Kamloops or Kawartha Lakes or Kelowna or Kingston or Kitchener or Langley or Laval or Lethbridge or Levis or Longueuil or Maple Ridge or Markham or Medicine Hat or Milton or Mirabel or Mississauga or Moncton or Montreal or Nanaimo or New Westminster or Newmarket or Niagara Falls or Norfolk County or North Bay or North Vancouver or North Vancouver or Oakville or Oshawa or Ottawa or Peterborough or Pickering or Port Coquitlam or Prince George or Quebec City or Red Deer or Regina or Repentigny or Richmond or Richmond Hill or Saanich or Saguenay or Saint John or Saint-Hyacinthe or Saint-Jean-sur-Richelieu or Saint-Jerome or Sarnia or Saskatoon or Sault Ste Marie or Sherbrooke or St Albert or St Catharines or St John's or Strathcona County or Surrey or Terrebonne or Thunder Bay or Toronto or Trois-Rivieres or Vancouver or Vaughan or ((Cambridge or (Halifax or Hamilton or London or Victoria or Waterloo or Welland or Whitby or Windsor)) not (UK or Britain or United Kingdom or England or Australia)) or Whitehorse or Winnipeg or Wood Buffalo or Yellowknife).ti,ab,kw. (581698)

21 exp Canada/ (182016)

22 20 or 21 (581700)

23 19 and 22 (154)

**Embase Search Terms (2010 to 2023)**

1 undocumented immigrant/ or immigrant/ (20,580)

2 emigrant/ (498)

3 refugee/ (16,455)

4 long distance migrant/ or migrant worker/ or forced migrant/ or migrant/ or short distance migrant /(14,107)

5 (immigrant* or immigration or emigrant* or emigration or refugee* or "asylum seeker*" or asylee* or "displaced person*" or "displaced people" or "incomer*" or "in comer*" or "new comer*" or newcomer* or migrant* or resettler*).mp.(100,536)

6 1 or 2 or 3 or 4 or 5 (100,536)

7 postcoitus contraceptive agent/ or emergency contraception/ or levonorgestrel/ (17,147)

8 vagina contraception/ or barrier contraception/ or contraception/ or long-acting reversible contraception/ or hormonal contraception/ or oral contraception/ (64,080)

9 family planning/ (38,682)

10 birth control/ (4,579)

11 "condom use"/ or condom/ or female condom/ (28,381_

12 medroxyprogesterone acetate/ (18,775)

13 norethisterone enantate/ (698)

14 estradiol valerate plus norethisterone enantate/ (94)

15 estradiol cipionate plus medroxyprogesterone acetate/ (12)

16 intrauterine contraceptive device/ (18,604)

17 vasectomy/ (5,467)

18 uterine tube ligation/ (3,565)

19 vagina ring/ (2,552)

20 abstinence/ (11,514)

21 (NET EN or "intrauterine system" or "intra-uterine system" or IUS or "intrauterine device" or "intra-uterine device" or IUD or sterilization or "tubal ligation" or "vaginal ring" or cycletel or "cycle-tel" or abstain or abstinen* or "lactational amenorr*").mp. (110,277)

22 sexual health/ or sexual behavior/ (132,185)

23 health services accessibility.mp. or health care access/ (85,864)

24 health care access/ or primary medical care/ or health equity/ or universal health care/ or " patient attitude "/ (292,446)

25 sexual health.mp. (29,765)

26 family planning.mp. (45,173)

27 birth control.mp. (8,119)

28 or/7-27 (636,279)

29 adolescent/ or adolescent pregnancy/ or adolescent/ or adolescent mother/ or adolescent father/ or adolescent parent/ or adolescent health/ or adolescent sexual behavior/ 1,770,000

30 child health care/ (38,513)

31 (middle school* or pubescen* or juvenile* or teen* or youth* or high school* or adolesc* or pre-pubesc* or prepubesc* or young adult*).mp. (2,460,119)

32 29 or 30 or 31 (2,486,740)

33 6 and 28 and (321,858)

34 exp Canada/ (215,590)

35 canada.mp. or exp Canada/ (286,871)

36 (Canad* or British Columbia or Colombie Britannique or Alberta* or Saskatchewan or Manitoba* or Ontario or Quebec or (New Brunswick not New Jersey) or Nouveau Brunswick or Nova Scotia or Nouvelle Ecosse or Prince Edward Island or Newfoundland or Labrador or Nunavut or NWT or Northwest Territories or Yukon or Nunavik or Inuvialuit).mp. (379,381)

37 34 or 35 or 36 (379,395)

38 33 and 37 (137)

**CINAHL Search Terms (2010 to 2023)**

| S1 | (MH "Immigrants+") OR(MH "UndocumentedImmigrants") OR (MH"Emigration andImmigration") | 23,991 |
| --- | --- | --- |
| S2 | (MH "Transients andMigrants") | 5,889 |
| S3 | immigrant* or immigrationor emigrant* or emigrationor refugee* or "asylumseeker*" or asylee* or"displaced person*" or"displaced people" or"incomer*" or "in comer*"or "new comer*" ornewcomer* or migrant* orresettler* | 46,387 |
| S4 | S1 OR S2 OR S3 | 46,387 |
| S5 | (MH "Contraception+") OR(MH "HormonalContraception") OR (MH"Contraceptives,  Postcoital+") OR (MH"Contraceptives, OralCombined") OR (MH"Contraceptive Agents,Male") OR (MH"Contraceptive Agents+")OR (MH "Diaphragms,Contraceptive") OR (MH"Contraceptive Devices+")OR (MH "IntrauterineDevices") OR (MH "Long-Acting ReversibleContraceptives") OR (MH"Contraceptive Agents,Hormonal+") OR (MH"Contraceptives, Oral+") | 43,992 |
| S6 | (MH "Family Planning+")OR (MH "Family PlanningPolicy") OR (MH "FamilyPlanning, Natural") | 13,577 |
| S7 | (MH "Sexual Health") | 8,526 |
| S8 | (MH "Sexual Behavior+") | 52,424 |
| S9 | (MH "Health ServicesAccessibility+") | 105,007 |
| S10 | (MH "Universal HealthCare") | 708 |
| S11 | (MH "Patient Attitudes") | 59,080 |
| S12 | (contracept* or "familyplanning" or "birth control"or condom or "depotmedroxyprogest*" or NET-EN or NET EN orMesigyna or Cyclofem or"intrauterine system" or"intra-uterine system" orIUS or "intrauterinedevice" or "intra-uterinedevice" or IUD or vasectomy or sterilizationor "tubal ligation" or"vaginal ring" or cycletel or"cycle-tel" or abstain orabstinen* or "lactationalamenorr*" | 78,071 |
| S13 | sexual health | 19,829 |
| S14 | family planning | 12,532 |
| S15 | health service* access* | 108,417 |
| S16 | universal health care | 2,726 |
| S17 | S5 OR S6 OR S7 OR S8OR S9 OR S10 OR S11OR S12 OR S13 OR S14OR S15 OR S16 | 306,195 |
| S18 | (MH "AdolescentParents") OR (MH"Adolescent Mothers") OR(MH "Adolescent HealthServices") OR (MH"Adolescent Fathers") OR(MH "AdolescentMedicine") OR (MH"Adolescent Health") OR(MH "Adolescence") OR  (MH "Maternal Age 14 andUnder") OR (MH"Pregnancy inAdolescence") | 602,779 |
| S19 | middle school* orpubescen* or juvenile* orteen* or youth* or highschool* or adolesc* or pre-pubesc* or prepubesc* oryoung adult* | 824,532 |
| S20 | S18 OR S19 | 824,544 |
| S21 | S4 AND S17 AND S20 | 1,733 |
| S22 | (MH "Canada+") | 111,838 |
| S23 | Canad* or British Columbia or Colombie Britannique or Alberta* or Saskatchewan or Manitoba* or Ontario or Quebec or (New Brunswick not New Jersey) or Nouveau Brunswick or Nova Scotia or Nouvelle Ecosse or Prince Edward Island or Newfoundland or Labrador or Nunavut or NWT or Northwest Territories or Yukon or Nunavik or Inuvialuit).mp, jw, nw. or (Abbotsford or Airdrie or Ajax or Aurora or Barrie or Belleville or Blainville or Brampton or Brantford or Brossard or Burlington or Burnaby or Caledon or Calgary or Cape Breton or Chatham Kent or Chilliwack or Clarington or Coquitlam or Drummondville or Edmonton or Fredericton or Fort McMurray or Gatineau or Granby or Grande Prairie or Sudbury or Guelph or Halton Hills or Iqaluit or Inuvik or Kamloops or Kawartha Lakes or Kelowna or Kingston or Kitchener or Langley or Laval or Lethbridge or Levis or Longueuil or Maple Ridge or Markham or Medicine Hat or Milton or Mirabel or Mississauga or Moncton or Montreal or Nanaimo or New Westminster or Newmarket or Niagara Falls or Norfolk County or North Bay or North Vancouver or North Vancouver or Oakville or Oshawa or Ottawa or Peterborough or Pickering or Port Coquitlam or Prince George or Quebec City or Rivieres or Vancouver or or Red Deer or Regina or Repentigny or Richmond or Richmond Hill or Saanich or Saguenay or Saint John or Saint-Hyacinthe or Saint-Jean-sur-Richelieu or Saint-Jerome or Sarnia or Saskatoon or Sault Ste Marie or Sherbrooke or St Albert or St Catharines or St John's or Strathcona County or Surrey or Terrebonne or Thunder Bay or Toronto or Trois-Rivieres or Vancouver or Vaughan or ((Cambridge or (Halifax or Hamilton or London or Victoria or Waterloo or Welland or Whitby or Windsor)) not (UK or Britain or United Kingdom or England or Australia)) or Whitehorse or Winnipeg or Wood Buffalo or Yellowknife | 243,534 |
| S24 | S22 OR S23 | 243,532 |
| S25 | S21 AND S24 | 153 |

**Web of Science Search Terms (2010 to 2023)**

| 1 | "emigrants and immigrants" OR "undocumented immigrants" OR "Refugees" OR "Transients and Migrants""immigrant" OR "immigration" OR "emigrant"" OR "emigration" OR "refugee" OR "asylum seeker*" OR "asylee"" OR "displaced person*" OR "displaced people" OR "incomer*" OR "in comer*" OR "new comer*" OR "newcomer*" OR "migrant*" OR "resettler*" | 175,477 |
| --- | --- | --- |
| 2 | TS=(contracept* or "family planning" or "birth control" or condom or "depot medroxyprogest*" or NET-EN or NET EN or Mesigyna or Cyclofem or "intrauterine system" or "intra-uterine system" or IUS or "intrauterine device" or "intra- uterine device" or IUD or vasectomy or sterilization or "tubal ligation" or "vaginal ring" or cycletel or "cycle-tel" or abstain or abstinen* or "lactational amenorr*" | 336,300 |
| 3 | TS=("middle school" or pubescen* or juvenile* or teen" or youth" or "high school" or adolesc* or pre-pubesc* or prepubesc* or "young adult*") | 1,096,280 |
| 4 | TS=((Canad* or British Columbia or Colombie Britannique or Alberta* or Saskatchewan or Manitoba* or Ontario or Quebec or (New Brunswick not New Jersey) or Nouveau Brunswick or Nova Scotia or Nouvelle Ecosse or Prince Edward Island or Newfoundland or Labrador or Nunavut or NWT or Northwest Territories or Yukon or Nunavik or Inuvialuit).mp,jw,nw. or (Abbotsford or Airdrie or Ajax or Aurora or Barrie or Belleville or Blainville or Brampton or Brantford or Brossard or Burlington or Burnaby or Caledon or Calgary or Cape Breton or Chatham Kent or Chilliwack or Clarington or Coquitlam or Drummondville or Edmonton or Fredericton or Fort McMurray or Gatineau or Granby or Grande Prairie or Sudbury or Guelph or Halton Hills or Iqaluit or Inuvik or Kamloops or Kawartha Lakes or Kelowna or Kingston or Kitchener or Langley or Laval or Lethbridge or Levis or Longueuil or Maple Ridge or Markham or Medicine Hat or Milton or Mirabel or Mississauga or Moncton or Montreal or Nanaimo or New Westminster or Newmarket or Niagara Falls or Norfolk County or North Bay or North Vancouver or North Vancouver or Oakville or Oshawa or Ottawa or Peterborough or Pickering or Port Coquitlam or Prince George or Quebec City or Rivieres or Vancouver or or Red Deer or Regina or Repentigny or Richmond or Richmond Hill or Saanich or Saguenay or Saint John or Saint-Hyacinthe or Saint-Jean-sur-Richelieu or Saint-Jerome or Sarnia or Saskatoon or Sault Ste Marie or Sherbrooke or St Albert or St Catharines or St John's or Strathcona County or Surrey or Terrebonne or Thunder Bay or Toronto or Trois-Rivieres or Vancouver or Vaughan or ((Cambridge or (Halifax or Hamilton or London or Victoria or Waterloo or Welland or Whitby or Windsor)) not (UK or Britain or United Kingdom or England or Australia)) or Whitehorse or Winnipeg or Wood Buffalo or Yellowknife)) | 385,590 |
| 5. | #1 AND #2 AND #3 AND #4 | 27 |
