# Supplementary material for: Newcomer youth’s access to contraception care in Canada: A scoping review of qualitative evidence
Source: PLoS One. 2025 Aug 4;20(8):e0327997. doi: 10.1371/journal.pone.0327997 (PMC12321124; doi:10.1371/journal.pone.0327997)
Supplement: S3 File — (DOCX) [file pone.0327997.s003.docx]

| No. | Article | Reason for exclusion |
| --- | --- | --- |
| 1 | Salehi R, Flicker S, Travers R, Larkin J, Flynn S, Layne C, Guta A. Predictors of exposure to sexual health education among teens who are newcomers to Canada. Can J Hum Sex. 2010;19(4):157-167. Ref ID: 361633069. | Quantitative study |
| 2 | Jarvis K, Farrell A, Richter S, Lukewich J. Determinants of accessibility to primary sexual reproductive health care among vulnerable women in Canada: A scoping review protocol. JBI Evid Synth. 2020;18(9):2065-2074. DOI: 10.11124/JBISRIR-D-19-00262. Ref ID: 634288922. | Study protocol |
| 3 | Salehi R, Hynie M, Flicker S. Factors associated with access to sexual health services among teens in Toronto: Does immigration matter? J Immigr Minor Health. 2014;16(4):638-645. DOI: 10.1007/s10903-013-9961-y. | Quantitative study |
| 4 | Hawkey AJ, Ussher JM, Perz J. What do women want? Migrant and refugee women's preferences for the delivery of sexual and reproductive healthcare and information. Ethn Health. 2022;27(8):1787-1805. DOI: 10.1080/13557858.2021.1980772. | Adult population |
| 5 | Pole JD, Flicker S, Flynn S, Layne C, Larkin J, Travers R, et al. Sexual behaviour profile of a diverse group of urban youth: An analysis of the Toronto Teen Survey. Can J Hum Sex. 2010;19(4):145-156. Ref ID: 361633068. | Quantitative study |
| 6 | Yu J. Young people of Chinese origin in western countries: a systematic review of their sexual attitudes and behaviour. Health Soc Care Community. 2010;18(2):117-28. DOI: 10.1111/j.1365-2524.2009.00906.x. Ref ID: 20459527. | Study protocol |
| 7 | Salehi R. Indicators of access to sexual health services for Toronto newcomer youth [dissertation]. Dissertation Abstracts International: Section B: The Sciences and Engineering. 2012;72(9-B):5253-5253. Ref ID: 2012-99060-283. | Quantitative study |
| 8 | Aptekman M, Rashid M, Wright V, Dunn S. Unmet contraceptive needs among refugees. Can Fam Physician. 2014;60(12):e613-9. Ref ID: 25642489. | Quantitative study |
| 9 | Dunn S, Xiong AQ, Nuernberger K, Norman WV. Non-use of contraception by Canadian youth aged 15 to 24: findings from the 2009-2010 Canadian Community Health Survey. J Obstet Gynaecol Can. 2019;41(1):29-37. DOI: 10.1016/j.jogc.2018.05.021. Ref ID: 30316712. | Quantitative study |
| 10 | Homma Y, Saewyc EM, Wong ST, Zumbo BD. Sexual health and risk behaviour among East Asian adolescents in British Columbia. Can J Hum Sex. 2013;22(1):13-24. Ref ID: 27087776. | Quantitative study |
| 11 | Shafiq F. Ensuring community university partnership to address HIV/AIDS and sexual reproductive health issues amongst newcomer African immigrant and refugee ethno-racial youth in Western Canada. Can J Infect Dis Med Microbiol. 2011;22(Suppl SB):107B-108B. | Does not describe the experiences of newcomer youth |
| 12 | Flicker S, Travers R, Flynn S, Larkin J, Guta A, Salehi R, et al. Sexual health research for and with urban youth: The Toronto Teen Survey story. Can J Hum Sex. 2010;19(4):133-144. | Does not describe the experiences of newcomer youth |
