# Supplementary material for: Newcomer youth’s access to contraception care in Canada: A scoping review of qualitative evidence
Source: PLoS One. 2025 Aug 4;20(8):e0327997. doi: 10.1371/journal.pone.0327997 (PMC12321124; doi:10.1371/journal.pone.0327997)
Supplement: Attachment — Submitted filename: Khan_Response to Editor and Reviewer_28 May 2024.pdf [file pone.0327997.s005.pdf]

Appeal: PLOS ONE Decision: PONE-D-24-05128

Response to Editor and Reviewer's Comments

Thank you for providing us with the opportunity to respond to the comments that we received from the editor and the peer reviewers. We have addressed the concerns raised by the editor, as well as provided responses to the minor revisions suggested by peer reviewers 1 and 2.

We received the following comment from the editor:

> *'The primary concern with this paper is the rationale behind exclusively incorporating qualitative studies for a scoping review. While a scoping review aims to map out and summarize existing literature on a specific topic, offering an overview of available evidence, it would be more comprehensive if quantitative studies were also included. Examining access to contraception using various study designs would allow for a more thorough exploration of research gaps and better inform policy and practice. Additionally, it is noteworthy that the authors only utilized four databases and limited the search for grey literature to published theses. Conducting a more comprehensive search is advisable for a scoping review.'*

We have provided a detailed rationale for conducting a scoping review of qualitative studies in our response to #3 and #4 of the minor comments (see below).

In addition, we wish to highlight that we searched the **five** indexed databases (Medline, Embase, CINAHL, PsycINFO and Web of Science). In addition, we also searched Theses Canada and UBC Library archives for relevant literature for published theses on this topic. We consulted a Knowledge Synthesis & Medical Librarian at the University of British Columbia (Vanessa Kitchin, MI) to identify relevant databases for this review, create the keyword search strategy, and run our scoping review searches.

We list the five databases in the PRISMA diagram that we submitted with our original manuscript. We had incorrectly classified this as four databases in the manuscript. We have amended this detail in the description of the data sources in the 'Abstract' and under the heading 'Sources'. These changes correspond to lines and lines 29 to 30, and lines 130 to 135 in the updated manuscript. The changes we made to the 'Sources' are also highlighted below.

> *'To include all relevant literature on this topic, we searched ~~four~~ five relevant databases (MEDLINE, Embase, CINAHL, PsycINFO, Web of Science) for indexed studies on June 14, 2023. We limited the grey literature to published theses to capture any recent literature that had not yet been published in scientific journals. Thesis searches were run in ~~Web of Science~~, Theses Canada, and UBC Library archives for relevant literature.'*

Below we provide a point-by-point response to the minor comments made by the editor:

1. For clarity, the abstract should explicitly mention the results of the five studies in the scoping review.

We revised the reporting of our results in the abstract to explicitly describe the findings of the five studies included in this review.

'Results: We screened 415 titles and abstracts and retrieved 17 eligible studies for full-text review, of which five were included for analysis. Results highlight that sociocultural influences play an important role in newcomer youth's perspectives and access to contraception care. The language preferences of newcomer youth in the context of contraception are unique and differ from the needs of the broader immigrant population seeking general health care. Results also identify formal and peer-based educational SRH interventions acceptable to newcomer youth.'

2. In the introduction, it is essential to present the latest data when discussing demographic trends in Canada.

The demographic data presented in our manuscript are from the 2021 Census conducted by Statistics Canada. As the census is conducted every five years, this represents the latest demographic data available. We have reviewed recent reports from Statistics Canada for relevant information. Although there have been newer reports, they continue to utilize data from the 2021 Census. Additionally, we have provided more recent demographic data about international students, as highlighted in red:

'Similar trends of growth have been documented among non-permanent residents in Canada. The 2021 census data enumerated 924,850 non-permanent residents, which includes international students, individuals on temporary work permits and asylum seekers [11]. Among non-permanent residents, 39.3% were 0 to 25 years old [11]. The growing number of newcomer youth in Canada underscores the importance of addressing their unmet needs for contraception care.'

3. The rationale for conducting this systematic review needs clarification. Authors should justify why a scoping review is chosen and why qualitative studies are exclusively focused on.

The objective of this systematic review was to inform the scope, design, and inclusion criteria for future qualitative research involving newcomers and contraception access in Canada. We were specifically interested at mapping gaps in the existing literature for this defined research area. We selected a scoping review as it aligned best with our objective. A scoping review is a "form of knowledge synthesis that addresses an exploratory research question aimed at mapping key concepts, types of evidence, and gaps in research related to a defined area or field by systematically searching, selecting, and synthesizing existing knowledge."[1] We have specified this at the end of the Introduction and in the beginning of the Methods, under Study Design.

'The purpose of this scoping review is to summarize the current qualitative evidence of newcomer youth's experiences with accessing contraception care and highlight gaps in the evidence. Our aim was to inform the scope, design, and inclusion criteria for future qualitative research involving newcomers and contraception access in Canada.'

'We followed the Joanna Briggs Institute (JBI) guidelines [12] to conduct a scoping review of published indexed literature and published theses. We selected a scoping review as it aligned best with our objective to map the knowledge and knowledge gaps in the existing qualitative research involving newcomers and contraception access in Canada.'

---

[1]Colquhoun H. Current best practices for the conduct of scoping reviews. 2016. Available from: http://www.equator-network.org/wp-content/uploads/2016/06/Gerstein-Library-scopingreviews_May-12.pdf

Regarding the rationale for our focus on the qualitative literature, youth experiences of accessing sexual and reproductive health (SRH) – including their attitudes, beliefs, preferences, and needs – can be nuanced, detailed, and difficult to quantify. In this regard, qualitative studies are best suited to investigate what youth are thinking, and how they are feeling when accessing SRH, and why they access or do not access care depending on context. Considering the objective and scope of our research, we focused on studies that used various qualitative data collection methods (focus groups, individual interviews, qualitative surveys, and arts-based methods).

4. The discussion highlighted the scarcity of published literature on the topic. Therefore, the authors should have included multiple study designs, as this aligns with the purpose of a scoping review rather than focusing solely on one study design.

Following the Joanna Briggs Institute guidelines, "*a scoping review can include any and all types of literature (eg, primary research studies, systematic reviews, meta-analyses, letters, guidelines, websites, blogs). However, reviewers may wish to impose limits based on the knowledge that particular types of sources would be most useful and appropriate.*"[2]

We followed this guidance to impose limits on knowledge sources that would be most useful and appropriate for addressing our research objective. Specifically, we limited our scoping review to include the most appropriate research studies, i.e., qualitative research, to address our objective, which was to summarize the evidence on newcomer youth's experiences with accessing contraception care to inform the scope, design, and inclusion criteria for future qualitative research involving newcomers and contraception access in Canada. The scarcity of publications on this topic area is <u>an important finding</u> of our review, which was determined after the completion of the scoping review. The paucity of qualitative evidence on this topic does not warrant the inclusion of quantitative studies or other methodologies that do not meet our research objective.

We wish to highlight that we selected PLOS One for submission because the journal has published qualitative reviews, including a *qualitative scoping review* in 2023.[3] The study was a scoping review of qualitative studies investigating reproductive health knowledge, attitudes, and practices in a single jurisdiction, Rwanda. We reviewed this publication and considered the peer review comments. Given the publication of this article in 2023, we felt that the scope and aims of our manuscript would be an excellent fit for PLOS ONE.

<u>Response to Reviewer 2's comments</u>

1. As observed in the abstract, kindly identify the eligibility criteria used and the data extraction process. Also, please change and improve the formatting of the abstract, it should include the following sections: Objectives >> Design >> Data Sources >> Eligibility Criteria >> Data extraction and synthesis >> Results >> Conclusions. For your reference, please visit the link

---

[2] Peters M, Marnie C, Tricco A, Pollock D, Munn Z, Alexander L, McInerney P, Godfrey C, Khalil H. Updated methodological guidance for the conduct of scoping reviews. JBI Evidence Synthesis 18(10):p 2119-2126, October 2020. | DOI: 10.11124/JBIES-20-00167

[3] Buser JM, August E, Rana GK, Gray R, Tengera O, et al. (2023) Scoping review of qualitative studies investigating reproductive health knowledge, attitudes, and practices among men and women across Rwanda. PLOS ONE 18(3): e0283833. https://doi.org/10.1371/journal.pone.0283833

provided http://bmjopen.bmj.com/content/8/3/e019438

We have reformatted the abstract in the updated manuscript to include highlight these sections. We tracked the changes that we made to the abstract.

2. Keywords should be arranged in alphabetical order

The keywords have been re-arranged in alphabetical order.

3. I noticed the search strategy relied solely on keywords. It's generally recommended to combine keywords with MeSH terms (tw) for more focused results. To improve transparency, could you include the search strategy used for each database in the supplementary files? This would be helpful, including the initial search terms and the final results after removing duplicates.

Our search strategy includes both keywords and MeSH terms. We searched the database Medline using the Ovid interface, where MeSH terms are determined by a '/'. The MeSH terms used in this search can be found on lines 1-3, 6, 8-9, 14-15 and 21 in our Medline search strategy.

We have included an updated version of the search strategy (the file has been renamed as '**S2 Search Strategy**') which includes the search terms for Embase and CINAHL and Web of Science. We were not able to include the search term for PsycInfo as the search was conducted by our Knowledge Synthesis & Medical Librarian at the University of British Columbia (Vanessa Kitchin, MI), who is currently on maternity leave until April 2025. We have requested access to this file from UBC Library services.

4. To enhance transparency in line with the PRISMA 2020 Checklist, could you consider including a table in the supplementary files that details the 12 excluded studies after full-text screening? The table could list each study and the corresponding reason for exclusion. This would provide valuable insight into the selection process.

We have included an additional file, '**S3 Reasons for Excluding Studies**' with a table listing each study and the corresponding reason for exclusion.

5. What is the tool you used in your data extraction, analysis and synthesis? Could you please present and discuss the specific tool and present the data in your paper? Using certain tools for each selected study design can ensure the quality of evidence and reduce the risk of bias in the conduct of your study.

As described in our original submission, we used Covidence to manage independent title/abstract screening, full text screening, data extraction, and risk of bias assessment. We used Excel for our data charting tables to manage data extraction, analysis, and synthesis. We have clarified this under **"Screening, study selection, and data charting"** on lines 188-190.

> *'After two co-authors (BO, ZK) conducted the searches, all identified citations were included in Covidence, a web-based software developed by the Cochrane group to streamline evidence synthesis reviews[20]. We used Covidence to manage independent title/abstract screening, full text screening, data extraction, and risk of bias assessment, after which we used Excel spreadsheets to manage data extraction, analysis, and synthesis.'*

The JBI guidelines suggest the following regarding data extraction,

> *'Extraction of results for a scoping review should include extraction of all data relevant to inform the scoping review objective/s and question/s. Charting tables or forms may be used (see Appendix 10.1 for a template tool). A descriptive summary of the main results organized based on the review inclusion criteria must be included.'*[4]

Following this guidance, we adapted the JBI data charting table and used this table to extract relevant information from the selected studies. We previously described our data extraction process in the manuscript (now lines 199 to 204),

> *'We adapted relevant headings for our data charting table from the JBI guidelines and a scoping review on adolescent health [12,21]. The data charting table consisted of the following headings: study title, authors, study objectives, methods, context or setting, participant characteristics, data analysis, and author's conclusions. Data extraction of the included studies was completed by one team member (VP), while a second member (BO) reviewed the extractions, consistent with the JBI scoping review guidelines.'*

Similarly, we followed JBI guidelines to conduct a descriptive qualitative synthesis of the included studies. This process included reviewing the *'included studies and data charts to summarize the qualitative methods, characteristics of participants, and descriptions of youth engagement in the research process*'. We have described our analysis in lines 206 to 213 of the manuscript, under the heading 'Analysis and synthesis'. We did not employe any specific 'tool' in the analysis of the qualitative data.

6. The manuscript mentions a data charting table with specific headings outlined on lines 160-162, including study title, authors, objectives, methods, context, participant characteristics, data analysis, and authors' conclusions. However, the paper currently only presents a table listing the included studies. For improved transparency and to allow readers to fully grasp the data analysis process, including the data charting table or a similar table with the mentioned headings in the supplementary files would be beneficial. This would provide a more comprehensive picture of the data extraction and analysis.

We have included an additional file, **'S4 Data Charting Table**' with the requested information.

7. To improve the flow of information, consider combining the "Data" heading and its context onto the same page as the actual data content. This will prevent unnecessary page breaks and enhance readability. For optimal formatting, it's recommended to follow the specific guidelines outlined in the PLOS One criteria for the "Data" section. This will ensure a consistent and professional presentation of your data.

---

[4] Peters M, Marnie C, Tricco A, Pollock D, Munn Z, Alexander L, McInerney P, Godfrey C, Khalil H. Updated methodological guidance for the conduct of scoping reviews. JBI Evidence Synthesis 18(10):p 2119-2126, October 2020. | DOI: 10.11124/JBIES-20-00167

We do not have a heading named "Data" in the submitted manuscript. We require clarification on the reviewer's suggestion to combine the "Data" heading and its context onto the same page.

Response to Reviewer 1's Comments

We have made the following minor changes and tracked them on the updated manuscript:

27 Embase, CINAHL, PsycINFO). Of 415 identified [insert 'studies'] through the search process, five [delete 'studies'] were

28 included in this scoping review following screening for eligibility. We [replace 'report' with 'reported'] the findings of our

33 seeking general health care. We also [replace 'provide' with 'provided'] insights on formal and peer-based educational

34 interventions acceptable to newcomer youth. [replace 'Literature' with 'Literatures'] that qualitatively [replace 'describes' with 'describe'] newcomer

35 youth's experiences with contraception [replace 'is' with 'are'] scarce, and existing [replace 'literature' with 'literatures'] only[replace ' mentions' with 'mentioned']

39 youth [insert comma (,)] is required to better understand their experiences.

51 [delete 'different'] migrant populations in Canada’, Chowdhury and colleagues (2021) identified sexual

60 for primary care services [replace comma (,) with period (.)] [replace 'which' with 'This'] included SRH services and contraception care [delete comma (,)] due to a lack of

73 population in Canada is projected to continue to grow and [insert 'will'] make up [replace '29%' with '29.1%' as cited in the study] to 34% of the

81 Barriers to newcomer [insert 'youth’s'] access to contraception care may be attributed to factors such as cultural

82 beliefs, cost of accessing care, and systematic barriers such as insurance coverage, [delete 'which']

90 We followed the Joanna Briggs Institute [delete '[12]' and insert '(JBI)'] guidelines [insert '[12]'] to conduct a scoping review of

93 PRISMA-ScR. The JBI guidelines offer flexibility in the data analysis process [12]. We [replace 'report' with 'reported'] the

99 to published theses to capture any recent [replace 'literature with 'literatures'] that [replace 'had' with 'has'] not yet been published in scientific

122 published before 2010 [replace 'would' with 'will'] not provide an accurate depiction of the current experiences of

155 included studies [replace comma (,) with period (.)] [Capitalize the word 'after'] which [insert comma (,) and 'two authors (ZK, BO) conducted'] qualitative analysis, synthesis, and interpretation [insert period (.) and delete 'were conducted']

156 [delete '(ZK, BO).'] The senior author (SM) provided feedback and guidance throughout the research

163 was completed by one team member (VP), while a second [insert 'member'] (BO) reviewed the extractions,

199 strategies [replace comma (,) with period (.) and replace 'which' with 'This'] was followed by a [replace 'focus' with 'focused'] group discussion to explore youths’ [replace 'perceptions' with 'perception'] of

206 educators [delete 'who participated'] in this interactive workshop [24]. The authors used mixed methods

214 videoconferencing video conferencing platform [insert comma (,)] Zoom [25]. Following the data collection, the authors applied

219 minute individual interviews on the videoconferencing platform [insert comma (,)] Google Meet [replace comma (,) with period (.) and insert 'The authors'] again analyzed

229 Participating youths were newcomers and street-involved [replace comma (,) with period (.) and capitalize 'however'], the authors did not collect

234 participated in this study lived in a Canadian city[replace semicolon (;) with period (.) and capitalize 'the'] name of the city was not disclosed in the

237 [replace 'The ‘Sex Education by Theatre (SExT)' with 'The study Taylor et al., (2022) conducted'] included 19 participants, with 10 participants who

253 Venezuela, [replace 'Columbia' with 'Colombia'], Saudi Arabia, Malaysia, Kuwait, Ireland, and Kazakhstan [26].
Thirteen

263 newcomer youth engagement in the research process [insert references '[22, 25]'.

285 'I think they [youth] are aware [replace semicolon (;) with period (.)] I was aware as early as when I was in my early

287 condoms and all those things [replace semicolon (;) with period (.) and capitalize 'but' then insert comma (,)] I think that people just don’t seem to like

288 using them [replace semicolon (;) with period (.) and capitalize 'because'] even if you are a youth and you go into a pharmacy to go

289 and buy condoms [insert comma (,)] or even to a mall to go and buy condoms people are going

290 to look at you in some weird way [replace semicolon (;) with period (.) and capitalize 'if'] you meet an elderly person, you will be

299 person you are buying the condom [replace semicolon (;) with period (.) and capitalize 'why']? Because they think [replace 'you' with 'you’re'] gonna have

300 sex [replace semicolon (;) with period (.) and capitalize 'when'] I came here the first month, I went to a convenience shop to buy

301 something, and this guy just came in a loud voice, [Quote "I want the blue Trojan"], in

302 front of everybody [replace semicolon (;) with period (.) and capitalize 'like'] he said it so loudly, [Quote “I am buying a condom”, replace semicolon (;) with period (.) and capitalize 'you'] can

303 never see anyone doing that in Ghana. [capitalize 'that’s'] one huge difference [replace semicolon (;) with period (.) and capitalize 'the']

304 awareness is there [insert comma (,)] but the perception of someone going to buy a condom, you

311 'In Ghana everyone just thinks of condom, condom use, [delete duplicate 'condom use', replace semicolon (;) with period (.), capitalize 'it' , delete 'was' and replace with 'wasn’t']

312 [delete 'not'] until I got here that I learnt really about contraception and the after-

313 morning pill that they do here [replace semicolon (;) with period (.) and capitalize 'so'] I don’t think that there is enough

326 Most of the newcomer participants agreed that [replace 'of' with 'while'] SRH workshop facilitators do not have to be

327 of the same cultural background as them, [delete 'however,'] they should be familiar with the cultural

441 backgrounds in facilitating the SRH education [23]. Taylor et al.’s [insert year of publication '(2022)'] evaluation of a theatre-based

447 needs of immigrant populations in Canada, Chowdhury et al. [insert year of publication '(2021)'] identified that immigrants from

450 scoping review. Chowdhury et al. [insert year of publication '(2021)'] further suggest that access to information about

509 This scoping review describes the current evidence on newcomer [replace 'youth' with 'youth’s'] experiences of

514 parental attitudes toward contraception care intersect and influence newcomer [replace 'youth' with 'youth’s'] access.

527 these research gaps can help policymakers, educators, and [Kindly spell out 'HCPs' as this was not previously mentioned] develop programs, design

577 Paediatr Child Health. 2019;24: [correct page '160–164' to '160-169']. doi:10.1093/pch/pxz033
