# Supplementary material for: Newcomer youth’s access to contraception care in Canada: A scoping review of qualitative evidence
Source: PLoS One. 2025 Aug 4;20(8):e0327997. doi: 10.1371/journal.pone.0327997 (PMC12321124; doi:10.1371/journal.pone.0327997)
Supplement: Attachment — Submitted filename: Comment.docx [file pone.0327997.s006.docx]

**Comment**

1. The result of this report is published at the University of Columbia as a thesis report. So, it is not original report.
2. Key words should be related to the research title e.g Emigrants
3. Youth refers to individuals aged 15 to 25 years. **What is your source/reference?**
4. Your study participants are between aged 15-25 years. You include the study conducted in Ghana by Boafo- Arthur (2013) which conducted on participants between aged 18-27 years which has difference in age. So, why you include? And how can draw conclusion from it? Clearly state it.
5. Another gap I will found in this study was current literature on healthcare access for immigrant populations identify language as a critical barrier for newcomers who do not speak English as their first language [27]. The results of our scoping review provide additional insights into the language preferences of youth specifically regarding SRH topics. Newcomer youth who spoke English as a second language expressed a preference for discussing SRH topics in English. How?
6. Conclusion should be based on your research question.
7. Technically the way they are planning for the teams are good for the contribution of the knowledge and skills in the study to assure the soundness of the finding.
