# Supplementary material for: Newcomer youth’s access to contraception care in Canada: A scoping review of qualitative evidence
Source: PLoS One. 2025 Aug 4;20(8):e0327997. doi: 10.1371/journal.pone.0327997 (PMC12321124; doi:10.1371/journal.pone.0327997)
Supplement: Attachment — Submitted filename: Response to Reviewers_4 June 2025.docx [file pone.0327997.s007.docx]

**Responses to ‘Edits Requested’ June 3, 2025**

We've checked your submission and before we can proceed, we need you to address the following issues:

1. We note that the grant information you provided in the ‘Funding Information’ and ‘Financial Disclosure’ sections do not match. When you resubmit, please ensure that you provide the correct grant numbers for the awards you received for your study in the ‘Funding Information’ section.

The correct grant numbers are included in the Funding Information section. We anticipate this comment refers to the two instances where “2021” is indicated in the Financial Disclosure (cited in comment 2 below). This refers to the year the funding was disbursed and is not an award number. We received different grants from each funder in subsequent years. The Funding Sources List in the Funding Information section references the correct Award Numbers for each grant.

2. Thank you for stating the following financial disclosure:
[ZK is supported by the Affiliated Scholarship at the University of British Columbia, Mitacs Fellowship, the Systems Change Grant (2021) from the Vancouver Foundation and the Community and University Engagement Support Funds (2021) from the University of British Columbia. SM is supported by a Michael Smith Health Research BC Scholar Award (18270)].
Please state what role the funders took in the study. If the funders had no role, please state: ""The funders had no role in study design, data collection and analysis, decision to publish, or preparation of the manuscript.""
If this statement is not correct you must amend it as needed.
Please include this amended Role of Funder statement in your cover letter; we will change the online submission form on your behalf.

**The funders had no role in study design, data collection and analysis, decision to publish, or preparation of the manuscript.** We have amended this in our cover letter. Thank you for changing the online submission form on our behalf.


3. Thank you for stating the following in the Competing Interests section:
[ZK is a board member of Options for Sexual Health (charity) and the Director of Free Periods Canada (registered non-profit society). ].

Please confirm that this does not alter your adherence to all PLOS ONE policies on sharing data and materials, by including the following statement: ""This does not alter our adherence to PLOS ONE policies on sharing data and materials.” (as detailed online in our guide for authors [https://urldefense.com/v3/__http://journals.plos.org/plosone/s/competing-interests__;!!K-Hz7m0Vt54!imAqNsL-GAiyy02kDzf4k_fEf0kYWHL5VPrtzfVjuR1JWqK1C66pO2WbDP_CTQaK--UJ_lyG7hxF81s$](https://urldefense.com/v3/__http:/journals.plos.org/plosone/s/competing-interests__;!!K-Hz7m0Vt54!imAqNsL-GAiyy02kDzf4k_fEf0kYWHL5VPrtzfVjuR1JWqK1C66pO2WbDP_CTQaK--UJ_lyG7hxF81s$) ). If there are restrictions on sharing of data and/or materials, please state these. Please note that we cannot proceed with consideration of your article until this information has been declared.

Please include your updated Competing Interests statement in your cover letter; we will change the online submission form on your behalf.


Co-author ZK’s competing interests **do not alter our adherence to PLOS ONE policies on sharing data and materials.** We have amended this in our cover letter. Thank you for changing the online submission form on our behalf.


4. We note that your Data Availability Statement is currently as follows: [All relevant data are within the manuscript.]

Please confirm at this time whether or not your submission contains all raw data required to replicate the results of your study. Authors must share the “minimal data set” for their submission. PLOS defines the minimal data set to consist of the data required to replicate all study findings reported in the article, as well as related metadata and methods ([https://urldefense.com/v3/__https://journals.plos.org/plosone/s/data-availability*loc-minimal-data-set-definition__;Iw!!K-Hz7m0Vt54!imAqNsL-GAiyy02kDzf4k_fEf0kYWHL5VPrtzfVjuR1JWqK1C66pO2WbDP_CTQaK--UJ_lyG1TukIsw$](https://urldefense.com/v3/__https:/journals.plos.org/plosone/s/data-availability*loc-minimal-data-set-definition__;Iw!!K-Hz7m0Vt54!imAqNsL-GAiyy02kDzf4k_fEf0kYWHL5VPrtzfVjuR1JWqK1C66pO2WbDP_CTQaK--UJ_lyG1TukIsw$) ).

For example, authors should submit the following data:

The values behind the means, standard deviations and other measures reported;
The values used to build graphs;
The points extracted from images for analysis.

Authors do not need to submit their entire data set if only a portion of the data was used in the reported study.

If your submission does not contain these data, please either upload them as Supporting Information files or deposit them to a stable, public repository and provide us with the relevant URLs, DOIs, or accession numbers. For a list of recommended repositories, please see [https://urldefense.com/v3/__https://journals.plos.org/plosone/s/recommended-repositories__;!!K-Hz7m0Vt54!imAqNsL-GAiyy02kDzf4k_fEf0kYWHL5VPrtzfVjuR1JWqK1C66pO2WbDP_CTQaK--UJ_lyGsObF5cI$](https://urldefense.com/v3/__https:/journals.plos.org/plosone/s/recommended-repositories__;!!K-Hz7m0Vt54!imAqNsL-GAiyy02kDzf4k_fEf0kYWHL5VPrtzfVjuR1JWqK1C66pO2WbDP_CTQaK--UJ_lyGsObF5cI$) .

If there are ethical or legal restrictions on sharing a de-identified data set, please explain them in detail (e.g., data contain potentially sensitive information, data are owned by a third-party organization, etc.) and who has imposed them (e.g., an ethics committee). Please also provide contact information for a data access committee, ethics committee, or other institutional body to which data requests may be sent. If data are owned by a third party, please indicate how others may request data access.

**All relevant data and methods are within the manuscript and the Supplementary Files.** This is a scoping review of qualitative literature. There are no quantitative means, standard deviations, other measures, graphs, etc. in this manuscript. The search strategy and data extraction methods and forms are included in our files, making it possible for anyone to replicate the results of our study.


5. Please include captions for your Supporting Information files at the end of your manuscript, and update any in-text citations to match accordingly. Please see our Supporting Information guidelines for more information: [https://urldefense.com/v3/__http://journals.plos.org/plosone/s/supporting-information__;!!K-Hz7m0Vt54!imAqNsL-GAiyy02kDzf4k_fEf0kYWHL5VPrtzfVjuR1JWqK1C66pO2WbDP_CTQaK--UJ_lyG0rp8e1w$](https://urldefense.com/v3/__http:/journals.plos.org/plosone/s/supporting-information__;!!K-Hz7m0Vt54!imAqNsL-GAiyy02kDzf4k_fEf0kYWHL5VPrtzfVjuR1JWqK1C66pO2WbDP_CTQaK--UJ_lyG0rp8e1w$) .

We have added an intext caption for our S3 supporting information files. All others were included at the end of the manuscript (page 8) and referenced in text following journal guidelines:

- S1. PRISMA-ScR Checklist (page 5, line 104)
- S2. Search Strategy (page 6, line 120)
- S3. Reasons for Excluding Studies (added to page 8, line 153)
- S4. Data Charting Table (cited on page 10, line 178)

6. Please ensure that you refer to Tables 1 and 2 in your text as, if accepted, production will need this reference to link the reader to the Table.

The reference to Tables 1 is in text (p. 8, line 160) and we have added a reference to Table 2 (page 11, line 195).
